# Beeswax Nanoemulsion for Consolidation and Hydrophobization of Canvases

**DOI:** 10.1002/cplu.202500058

**Published:** 2025-05-27

**Authors:** Yiming Jia, Krister Holmberg, Romain Bordes

**Affiliations:** ^1^ Department of Chemistry and Chemical Engineering, Applied Chemistry Chalmers University of Technology 412 96 Gothenburg Sweden

**Keywords:** beeswax nanoemulsions, canvas consolidations, cellulose nanocrystals, cultural heritage conservation, Ouzo effect

## Abstract

In this article, a novel dispersion system is presented for consolidation and hydrophobization of degraded canvases. The dispersions are formulated by combining cellulose nanocrystals (CNC) and hydrophobically modified ethylhydroxyethylcellulose (EHM) with beeswax nanoemulsions. The beeswax nanoemulsion is prepared using the Ouzo effect, a low‐energy, surfactant‐free, and spontaneous emulsification method. EHM serves as an anchor for CNC and beeswax nanoparticles on the canvas, forming a continuous film and preventing aggregation during the treatment process. Tensile tests and contact angle measurements demonstrate that the dispersions effectively strengthen the canvas and enhance its water resistance, addressing the moisture‐induced mechanical limitations observed with previous nanocellulose and polyelectrolyte consolidants. Additionally, the hydrophobicity of the beeswax‐CNC/EHM system can be adjusted by varying the beeswax content. Overall, the use of entirely green components and the straightforward preparation process makes this dispersion system highly adaptable for consolidation in cultural heritage conservation, particularly in scenarios where a hydrophobic surface is required.

## Graphical abstract

A beeswax‐CNC/EHM composite system is developed for the consolidation and hydrophobization of canvases. The beeswax nanoemulsion is prepared using a spontaneous emulsification method based on the Ouzo effect. The interactions between CNC, EHM and beeswax resulted in a stable and uniform layer upon drying, synergistically providing both reinforcement and hydrophobization for the degraded canvases.

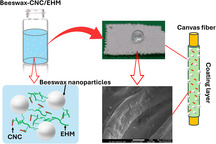

## 1 Introduction

Canvas degradation is primarily attributed to fluctuations in temperature and humidity, as well as chemical damage resulting from oxidation and acid‐catalyzed hydrolysis of cellulose fibers.^[^ ^1^, ^2^ ^]^ Consolidating and deacidifying the canvas using adhesives is considered a more efficient and less invasive alternative to traditional lining techniques, which have been widely employed in cultural heritage conservation.^[^ ^3^ ^]^

In this context, both natural and synthetic materials, such as animal glue, Beva 371, and acrylic resins, are commonly used to strengthen fragile painting canvases.^[^ ^4^ ^]^ Deacidification treatments typically involve the application of alkaline chemicals, like calcium hydroxide, magnesium bicarbonate, and magnesium methoxy methyl carbonate.^[^ ^5^ ^]^

Recent trends in art restoration emphasize minimal intervention, which has led to the increased use of nanoparticulate systems. Their small size and high surface area allow for effective application even at low concentrations. Various nanoparticles, including nanocellulose,^[^ ^6^, ^7^ ^]^ lignin nanoparticles,^[^ ^8^ ^]^ alkaline nanoparticles,^[^ ^9^, ^10^, ^11^ ^]^ and colloidal silica,^[^ ^12^, ^13^ ^]^ have been developed for strengthening and deacidifying degraded canvases.

Since canvas is a cellulose‐based material, the use of nanocellulose is highly compatible with the original substrate. However, nanocellulose's large aspect ratio tends to promote film formation on the canvas surface with limited penetration, resulting in weak adhesion.^[^ ^13^ ^]^ To address this issue, cationic and anionic polymers have been incorporated to create multilayered treatments that improve penetration and provide effective consolidation at different length scales.^[^ ^13^, ^14^ ^]^ Despite these improvements, the mechanical response of these materials to changes in moisture remains significant, whether applied alone or in combination with polyelectrolyte materials.^[^ ^6^, ^14^ ^]^

This sensitivity to relative humidity is detrimental to the long‐term consolidation of the canvas. Additionally, the variation in humidity caused by temperature changes can promote mold growth and exacerbate moisture‐induced damage.^[^ ^15^ ^]^

Waxes are among the most hydrophobic substances found in nature, composed primarily of long‐chain aliphatic compounds or long‐chain esters. They offer advantages, such as easy availability, cost‐effectiveness, and inherent water‐repelling properties. Waxes have been utilized in the paper and textile industries to create hydrophobic layers. However, textiles treated with solid wax films often face the drawback of reduced breathability. Recrystallization from solvents or emulsions has been proposed as a more effective approach to address this issue and has been applied in various fields.^[^ ^16^, ^17^, ^18^, ^19^, ^20^, ^21^, ^22^ ^]^

In nature, biomimetic superhydrophobic surfaces, such as those of lotus and rice leaves, are attributed to hierarchical surface structures with nanostructured hydrophobic epicuticular wax crystals.^[^ ^23^, ^24^ ^]^ Wax nanoemulsions offer hydrophobic nanoparticles that can be applied onto surfaces to mimic such structure, and can therefore be considered for canvas surfaces to mitigate the effects of humidity, providing a protective and water‐repellent layer.

Nanoemulsions, characterized by a higher interfacial area compared to macroemulsions, exhibit greater kinetic stability and are less prone to phase separation.^[^ ^25^ ^]^ There are various methods available for producing nanoemulsions, which can be categorized into high‐energy and low‐energy emulsification methods based on energy consumption.^[^ ^26^ ^]^ High‐energy methods rely on external energy inputs to break droplets, employing techniques, such as high‐shear stirring, high‐pressure homogenization, and ultrasonication. In contrast, low‐energy methods utilize the chemical potential of emulsion components to induce phase transitions, as seen in phase inversion temperature, phase inversion composition, and spontaneous emulsification methods.^[^ ^26^, ^27^ ^]^

Low‐energy methods offer advantages, such as lower energy consumption and greater stability of the nanoemulsion formed.^[^ ^28^ ^]^ However, compared to high‐energy methods, they typically require a higher concentration of surfactants to produce small nanodroplets, which limits their applicability.^[^ ^29^ ^]^ One notable exception is the Ouzo effect, a classic example of surfactant‐free, spontaneous emulsification. This method provides an appealing, straightforward, and energy‐efficient approach to prepare dispersions, particularly on an industrial scale.^[^ ^30^ ^]^ The process involves adding a small amount of oil dissolved in a water‐miscible solvent to water, resulting in spontaneous nanoemulsion formation.^[^ ^31^ ^]^ The order of mixing the ingredients does not critically affect the process.^[^ ^32^ ^]^ However, there is very little research reported on developing wax nanoemulsions using low‐energy methods.^[^ ^33^ ^]^ To the authors’ knowledge, no studies have explored the application of wax nanoemulsions prepared by low‐energy methods in cultural heritage conservation.

In this study, we present a novel combined approach utilizing CNC and beeswax nanoemulsion for consolidation and hydrophobization of canvases. The beeswax nanoemulsion was prepared by the Ouzo effect. To improve the adhesion of nanoparticles to the fiber surface, a noncharged cellulose‐based material, ethylhydroxyethylcellulose (EHM), was introduced. EHM is known for its ability to increase the viscosity of coatings formulations through self‐association and interaction with surfactant micelles.^[^ ^34^, ^35^ ^]^ Here, we use EHM to establish a network between CNC and beeswax nanoemulsion droplets, reinforcing the canvas while at the same time providing a water‐resistant protective layer.

## 2 Results and Discussion

**Figure 1:**
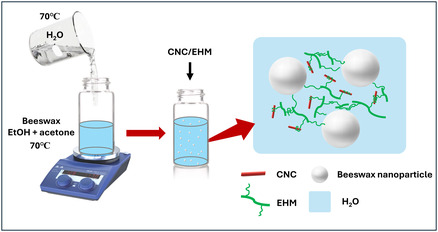
Schematic illustration of preparation and structure of the beeswax–CNC/EHM dispersions.

The green dispersion developed for the consolidation and hydrophobization of degraded canvases is shown in **Figure** **1**. EHM is a noncharged polymer which does not readily induce phase separation with negatively charged CNC and beeswax nanodroplets due to the absence of electrostatic interactions. However, gelation was observed when CNC and EHM were mixed (Figure S1, Supporting Information), suggesting the presence of physical interactions, likely driven by hydrophobic interactions between the hydrophobic side chains of EHM and the hydrophobic regions of CNC. EHM functions as a noncovalent cross‐linker, facilitating the integration of CNC and beeswax nanodroplets, while at the same time providing additional adhesion strength and the possibility of controlling the viscosity of the suspension. **Table** **1** shows the prepared formulations, including the weight percentages of each component. The dispersions were prepared with low component concentrations: the beeswax concentration ranged from 0.11% to 0.5% and the CNC/EHM concentration ranged from 0.5% to 0.89%. The total solids content of the dispersion remained below 3%. The appearance of the formulations is shown in Figure S1 (Supporting Information). Due to the interaction between EHM and CNC, formulations with higher CNC/EHM content, such as beeswax0.11–CNC/EHM0.89 and beeswax0.2–CNC/EHM0.8, exhibit a gel‐like texture. In contrast, reducing the CNC/EHM content, as in beeswax0.33–CNC/EHM0.67 and beeswax0.5–CNC/EHM0.5, results in more fluid dispersions, which are better suited for consolidation treatments.

**Table 1:** Formulation details for the dispersions.

Formulations	Dry mass content [wt%]	Dry mass composition [wt%]		
		Beeswax	CNC	EHM
Beeswax1a)	1.0	100.0	–	–
CNC/EHM2	2.0	–	50.0	50.0
Beeswax0.11–CNC/EHM0.89	3.0	11.0	44.5	44.5
Beeswax0.2–CNC/EHM0.8	2.5	20.0	40.0	40.0
Beeswax0.33–CNC/EHM0.67	2.0	33.0	33.5	33.5
Beeswax0.5–CNC/EHM0.5	1.6	50.0	25.0	25.0

a ^)^1.0 wt% beeswax nanoemulsion.

**Figure 2:**
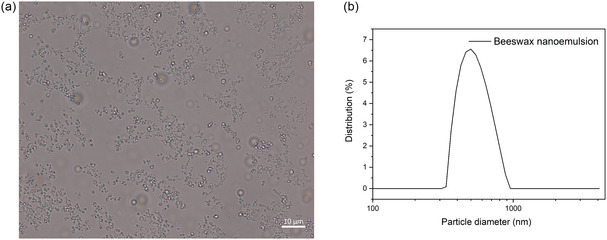
Optical microscopy with a) bright field and b) DLS analysis of beeswax nanoemulsion.

The appearance and size of the beeswax nanoparticles were initially investigated. **Figure** **2**a presents the optical microscopy image of the beeswax nanoemulsion, revealing that the dispersion consists of spherical nanoparticles. The size distribution of the droplets was analyzed using dynamic light scattering (DLS). As shown in Figure 2b, the beeswax dispersion exhibits a unimodal size distribution within the range of 300–950 nm, with an average droplet size of 540 nm. Additionally, the zeta potential analysis indicated a negative charge of −39 mV, confirming the stability of the beeswax nanoemulsion prepared via the Ouzo effect. The observed negative charges could be attributed to the presence of carboxyl groups in beeswax.^[^ ^36^ ^]^

**Figure 3:**
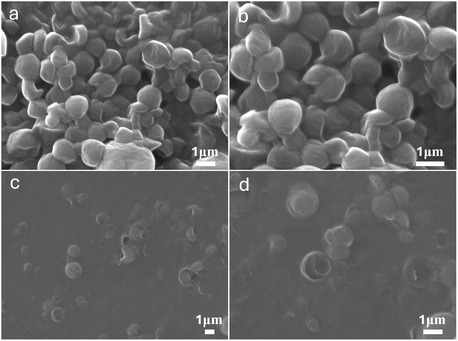
SEM image of dried a,b) beeswax nanoemulsion and c,d) beeswax0.5–CNC/EHM0.5.

Scanning electron microscopy (SEM) images of beeswax nanoparticles and the beeswax0.5–CNC/EHM0.5 composite are presented in **Figure** **3**. The dry beeswax nanoparticles are observed as nanospheres with some surface deformation. The deformed and broken nanospheres visible in the figure are believed to be due to their hollow structure. In the case of beeswax nanoemulsion alone, the nanoparticles tend to aggregate during the drying process, making it challenging to achieve a uniform coating layer. However, the addition of CNC/EHM effectively mitigates aggregation, enabling the formation of a more even and consistent coating layer.

**Figure 4:**
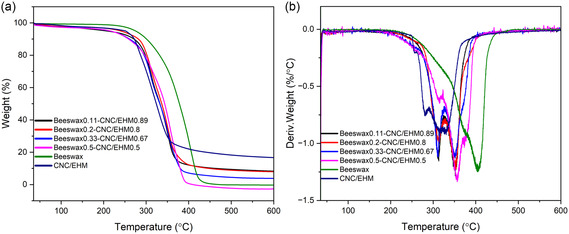
a) TGA and corresponding b) derivative thermogravimetry curves of beeswax0.11–CNC/EHM0.89 (black), beeswax0.2–CNC/EHM0.8 (red), beeswax0.33–CNC/EHM0.67 (blue), beeswax0.5–CNC/EHM0.5 (pink), beeswax (green), and CNC/EHM (purple).

Thermogravimetric analysis (TGA) was used to evaluate the thermal stability of the dried beeswax–CNC/EHM dispersion. For comparison, pristine components of CNC, EHM, beeswax, as well as the CNC/EHM mixture were also analyzed. As shown in Figure S2 (Supporting Information) and **Figure** **4**, the maximum degradation temperatures of pristine CNC, EHM, and beeswax were observed at 305, 363, and 405 °C, respectively. However, the CNC/EHM mixture exhibited significantly lower degradation temperatures at 258, 282, and 330 °C, indicating reduced thermal stability compared to individual components. This early degradation may be attributed to a reduction in crystallinity and the catalytic effect of sulfate groups present in CNC.^[^ ^37^ ^]^ The beeswax–CNC/EHM composites showed the maximum degradation temperatures around 310, 352, and 378 °C, which are obviously higher than the CNC/EHM composite. This improvement is likely due to the good compatibility among components, with beeswax acting as a thermal barrier. Nonetheless, the presence of acidic sulfate groups in CNC may still reduce the thermostability of beeswax, as evidenced by the TGA results. Overall, the beeswax–CNC/EHM composites exhibit commend thermal stability, showing significant improvement over the CNC/EHM composite.

**Figure 5:**
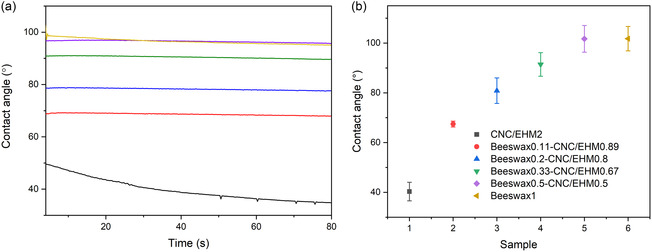
a) Contact angle as a function of time and b) average contact angle of the formulations and control samples applied on glass substrates.

The appearance of the formulations after drying on glass slides is shown in Figure S3 (Supporting Information). The wettability of the coating layers prepared from various formulations was evaluated by measuring contact angle of water droplets (**Figure** **5**). Coating layers composed solely of CNC/EHM exhibited high hydrophilicity, with a water contact angle of ≈40°. As anticipated, the incorporation of beeswax nanoparticles enhanced the hydrophobicity of the coating layer, with the contact angle remaining stable over time. Increasing the beeswax content in the dispersion from 0.11% to 0.5% resulted in an increase in the contact angle from 70° to 96°. When the beeswax content exceeded 0.3%, such as in the beeswax0.33‐CNC/EHM0.67 formulation, the coating layer was clearly hydrophobic. At higher beeswax concentrations, such as beeswax0.5–CNC/EHM0.5, a contact angle comparable to that of the pure beeswax nanoemulsion was obtained, ≈96°. However, the contact angle of the pure beeswax nanoemulsion was slightly lower than expected, most likely due to the low concentration of beeswax and to challenges in forming a uniform, continuous coating film during the drying process.

**Figure 6:**
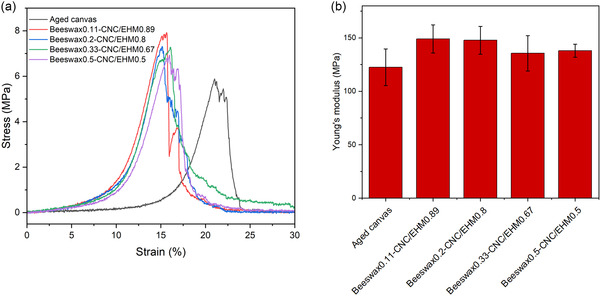
(a) Stress‐strain curves and (b) Young's modulus of the threads of the degraded cotton canvases before and after treatment with the formulations.

Tensile tests were performed to evaluate the strengthening capacity of the dispersions for canvas consolidation. The dispersions were applied to aged warp threads by brushing, and the resulting mechanical properties are summarized in **Table** **2**, with tensile stress–strain curves shown in **Figure** **6**a. The thread degradation process followed the previously described accelerated ageing method.^[^ ^38^ ^]^ After treatment, the threads exhibited an average weight increase of ≈2–3%. Untreated, degraded threads showed a maximum tensile strength of 5.94 MPa and an elongation at break of 21%. In comparison, threads coated with the beeswax0.11–CNC/EHM0.89 dispersion exhibited an increased tensile strength of 7.58 MPa, while the strain at break decreased to 15%, indicating that the consolidation enhanced the strength of the thread but reduced its stretchability.

**Table 2:** Mechanical properties of the degraded cotton canvas threads before and after treatment with the formulations.

Formulations	Tensile strength [MPa]	Strain at max. load [%]	Average mass increase [wt%]
No‐treatment	5.94 ± 0.82	20.58 ± 0.71	0
Beeswax0.11–CNC/EHM0.89	7.56 ± 0.75	15.20 ± 1.90	1.89
Beeswax0.2–CNC/EHM0.8	7.58 ± 0.64	15.60 ± 2.80	2.52
Beeswax0.33–CNC/EHM0.67	7.08 ± 0.80	15.83 ± 1.84	3.30
Beeswax0.5–CNC/EHM0.5	7.06 ± 0.47	16.30 ± 2.11	2.10

Young's modulus represents the material's stiffness. As shown in Figure 6b, the treated threads demonstrated higher Young's modulus values compared to the untreated ones, confirming an increase in stiffness following consolidation. However, as the beeswax content increased in the formulations, Young's modulus slightly decreased, suggesting enhanced flexibility in formulations with higher beeswax content. While different formulations exhibited minor variations in tensile strength and stiffness, the overall increase in tensile strength and Young's modulus indicates that a stiff protective layer is formed on the threads after treatment with the dispersions.

The wettability of the aged canvas before and after treatment with various dispersions was evaluated by measuring the water contact angle. The treated surfaces exhibited slow droplet spreading, which can be described by a quasi‐equilibrium spreading model: $y=y_{0}+A\times \exp\left(-x\times k\right)$. Here, *A* is a fitting parameter, *k* is an adjustable parameter related to spreading and reflects the required time to reach equilibrium, and *y*_0_ is the quasi‐equilibrium contact angle. In general, a smaller *k* value corresponds to a longer time to reach equilibrium.^[^ ^39^ ^]^

**Figure 7:**
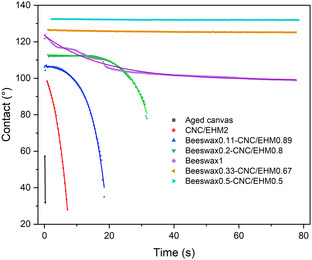
Contact angle as a function of time of the formulations and control samples applied on degraded canvas. The line is the fitting model discussed.

**Figure** **7** and Table S1 (Supporting Information) present the dynamic contact angle curves and the corresponding fitted results. The untreated degraded canvas was highly hydrophilic, with water droplets being absorbed immediately upon contact. In contrast, the treatment canvases showed two distinct wetting behaviors. Canvas treated with CNC/EHM, beeswax0.11–CNC/EHM0.89, and beeswax0.2–CNC/EHM0.8 exhibited negative values for both A and k, which do not conform to the standard spreading model. These surfaces initially resisted wetting, followed by sudden spreading, indicating a complex wetting mechanism involving both the consolidation layer and the underlying degraded canvas. Wettability tests on glass substrates confirmed that CNC/EHM is essentially hydrophilic. When applied to the porous, degraded canvas, the CNC/EHM coating rapidly swelled upon water contact. The combination of beeswax nanoparticles enhanced water resistance. Consolidants of beeswax0.11–CNC/EHM0.89 and beeswax0.2–CNC/EHM0.8 provided moderate water barriers; however, due to the canvas's porous structure, gradual water penetration still occurred and subsequently be absorbed by canvas.

Formulations with beeswax0.33–CNC/EHM0.67 and beeswax0.5–CNC/EHM0.5 exhibited typical quasi‐equilibrium spreading behavior, with very small *k* values of 0.034 and 0.056, respectively, indicating slow wetting process. These formulations maintained high contact angles of ≈120° and 130°, suggesting strong water‐repellent properties and effective barrier performance. Similarly, the pure beeswax nanoemulsion also showed slow spreading (*k* = 0.055), though with a lower contact angle of 100°, attributed to poor adhesion between the nanoemulsion and canvas fibers. This was further supported by a minimal weight increase of only 0.17% after application, compared to 2.5–5.9% observed in other formulations.

Therefore, the formulations of beeswax0.33–CNC/EHM0.67 and beeswax0.5–CNC/EHM0.5 demonstrated excellent hydrophobicity and strong resistance to spontaneous wetting, making them effective candidates for improving canvas water resistance.

The color change of the canvas resulting from the treatment with the dispersions was evaluated. As shown in **Table** **3**, the commission internationale de l'Éclairage color coordinates (L*, a*, b*) represent the color values in the color space, while ΔE* indicates the overall color change. Compared to the untreated degraded canvas, the color change for CNC/EHM2 was measured as 0.61 (ΔE*). With increasing beeswax content, ΔE* values rose to 0.61, 0.64, 1.05, and 1.56 for beeswax0.11–CNC/EHM0.89, beeswax0.2–CNC/EHM0.8, beeswax0.33–CNC/EHM0.67, and beeswax0.5–CNC/EHM0.5, respectively. In general, ΔE* values below 1.0 are considered imperceptible to the human eye, indicating no visible change. Therefore, treatments with dispersions containing beeswax nanoparticles up to 0.33% do not result in noticeable color changes.

**Table 3:** The color change of degraded cotton canvas before and after treatment with the formulations.

Formulations	L*	a*	b*	ΔE*
No‐treatment	89.59 ± 0.02	0.08 ± 0	1.72 ± 0.01	0
Beeswax1	90.74 ± 0.20	0	1.73 ± 0.06	1.15 ± 0.20
CNC/EHM2	90.13 ± 0.08	0 ± 0.02	2.01 ± 0.01	0.61 ± 0.07
Beeswax0.11–CNC/EHM0.89	90.06 ± 0.17	0.04 ± 0.04	2.10 ± 0.10	0.61 ± 0.16
Beeswax0.2–CNC/EHM0.8	89.95 ± 0.28	−0.02 ± 0.03	2.14 ± 0.23	0.64 ± 0.11
Beeswax0.33–CNC/EHM0.67	90.55 ± 0.25	−0.04 ± 0.02	2.13 ± 0.06	1.05 ± 0.21
Beeswax0.5–CNC/EHM0.5	91.15 ± 0.02	−0.01 ± 0.01	1.87 ± 0.04	1.56 ± 0.01

**Figure 8:**
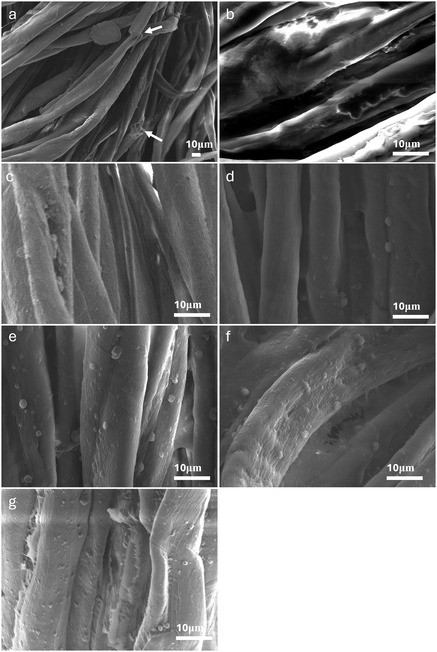
SEM image of degraded canvas a) before and after treatment with b) CNC/EHM2, c) beeswax1 d) beeswax0.11–CNC/EHM0.89, e) beeswax0.2–CNC/EHM0.8, f) beeswax0.33–CNC/EHM0.67, and g) beeswax0.5–CNC/EHM0.5.

The morphology of the canvas before and after application of the dispersions was examined with SEM observations. As shown in **Figure** **8**a, the untreated degraded cotton fibers exhibited visible ruptures and cracks on their surfaces. As shown in Figure 8b, after treatment with 2% CNC/EHM, a dense but discontinuous coating layer was observed on the fiber surfaces. This discontinuity is likely attributed to the gel‐like nature of CNC/EHM, which has low fluidity and thus difficulties in achieving uniform coverage on the canvas fibers.

Beeswax nanoparticles were visible on the fiber surfaces after applying formulations containing beeswax nanoemulsion (Figure 8c–g). However, when only the beeswax nanoemulsion was used, significantly fewer nanoparticles adhered to the fibers compared to the beeswax–CNC/EHM formulations, which is consistent with the mass increase after treatment (Table S2, Supporting Information). This observation indicates that it is the weak adhesion of pure beeswax nanoparticles that limits its effectiveness in improving water resistance. The beeswax–CNC/EHM formulations gave a uniform distribution on the fiber surfaces without agglomeration, with beeswax nanoparticles attached to the fibers. This confirms that EHM acted as an anchor for CNC and beeswax, exhibiting excellent compatibility with the canvas. Furthermore, this combination facilitated the formation of hierarchical surface structures with hydrophobic beeswax nanoparticles on the canvas fibers, mimicking natural superhydrophobic surfaces. SEM images at both low and high magnification (Figure 8 and Figure S4, Supporting Information, respectively) revealed that the formulations formed a thin, consistent coating layer on the canvas. This coating layer did not alter the macroscopic appearance of the canvas. Furthermore, the beeswax–CNC/EHM formulations maintained a significant portion of the porous structure of the canvas, ensuring that its breathability was preserved after treatment.

## 3 Conclusion

In this study, a novel green dispersion system combining CNC, beeswax nanoparticles, and EHM was developed for the consolidation and hydrophobization of degraded canvases. The beeswax nanoemulsion was prepared using a spontaneous emulsification method based on the Ouzo effect, which does not require surfactants. The properties of the beeswax nanoemulsion were characterized using optical microscopy, DLS, zeta potential analysis, and SEM. In this system, the interactions between EHM, CNC, and beeswax resulted in a stable and uniform layer after drying, synergistically achieving both reinforcement and hydrophobization of the degraded canvases. Wettability and tensile tests were conducted to evaluate the performance of the formulations, with beeswax0.33–CNC/EHM0.67 and beeswax0.5–CNC/EHM0.5 emerging as the most effective. The color change induced by the dispersions was nearly imperceptible to the human eye. The morphology of the degraded canvas after treatment revealed the formation of hierarchical surface structures with hydrophobic beeswax nanoparticles on the fibers, mimicking natural superhydrophobic surfaces. Moreover, the thin and consistent coating layer formed on the canvas fibers preserved the porous structure, maintaining the breathability of the canvas.

In summary, this study introduces an eco‐friendly, easy‐to‐prepare dispersion system for the consolidation and hydrophobization of aged canvases, offering significant potential for applications in cultural heritage conservation, particularly in cases requiring a hydrophobic surface.

## 4 Experiment Section

4.1

4.1.1

##### Materials

Cellulose nanocrystals (CNC) were obtained from CelluForce, Canada. Red ocher pigment and beeswax (bleached) were purchased from Kremer Pigmente GmbH & Co. Bermocoll EHM 200 (hydrophobically modified EHEC) was obtained from Nouryon, Sweden. Sulfuric acid (95–97%) was purchased from Sigma‐Aldrich, Sweden. Hydrogen peroxide (>30%) was purchased from Fisher Scientific, Sweden. Plain‐weave cotton canvas was purchased from BarnaArts (Barcelona, Spain).

##### Preparation of the Formulations

A 1% weight percent beeswax nanoemulsion was prepared by dissolving 300 mg of beeswax in a mixture of 7.35 g ethanol and 7.35 g acetone, stirred at 70 °C until fully dissolved. Subsequently, 15 g of prewarmed water (70 °C) was added to the beeswax solution under magnetic stirring at 800 rpm, which was then cooled to room temperature.

To prepare a 2% CNC/EHM dispersion, 400 mg of EHM was added to 19.6 g of a 2% CNC dispersion and stirred at room temperature.

The beeswax–CNC/EHM formulations were prepared as follows: Beeswax0.11–CNC/EHM0.89: 2 g of the 1% beeswax nanoemulsion was mixed with 4 g of the 2% CNC/EHM dispersion. Beeswax0.2–CNC/EHM0.8: 4 g of the 1% beeswax nanoemulsion was mixed with 4 g of the 2% CNC/EHM dispersion. Beeswax0.33–CNC/EHM0.67: 4 g of the 1% beeswax nanoemulsion was mixed with 2 g of the 2% CNC/EHM dispersion. Beeswax0.5–CNC/EHM0.5: 4 g of the 1% beeswax nanoemulsion was mixed with 1 g of the 2% CNC/EHM dispersion.

##### Accelerated Degradation of Painting Canvas

Accelerated degradation of the painting canvas was conducted following a previously reported chemical treatment method.^[^ ^38^ ^]^ Specifically, a 70 × 80 mm piece of new cotton canvas was immersed in 200 mL of hydrogen peroxide solution combined with 10 mL of sulfuric acid. The degradation process was performed at 40 °C for 72 h in an oven. The degraded canvas was thoroughly washed and dried under biaxial tension to prevent shrinkage. After drying, the warp threads were separated from the canvas for subsequent testing. The degradation process reduced the tensile load of a warp thread from 11.2 to 2.7 N and the strain at break from 30% to 20.6%.

##### Application of the Formulations

Glass samples were obtained by applying a 100 μm layer of the formulations onto glass slides using an applicator frame. Thread samples were obtained from the warp direction of artificially aged canvases. The formulations were applied to the surface of the degraded cotton threads using a brush. Canvas samples were obtained by immersing the 2 × 2 cm of degraded canvases in the formulations for 10 min. Excess consolidant was gently wiped from the surface before allowing the samples to dry. All the samples were dried at room temperature for 24 h before measurements.

##### Optical Microscopy

Microscopy images were obtained using a Carl Zeiss Axio Imager Z2m microscope under brightfield.

##### TGA

TGA was carried out using a TGA differential scanning calorimetry 3+ Stare instrument (Mettler Toledo). The thermograms were recorded from 30 to 600 °C at a heating rate of 10 °C min^−1^ under N_2_ atmosphere.

##### Dynamic Light Scattering and Zeta Potential Analysis

The particle size of the beeswax nanoemulsion was determined with a Litesizer 500 equipment (40 mW semiconductor laser of 658 nm). The beeswax nanoemulsion was diluted in Milli‐Q water and ultrasonicated for 1 min. The zeta potential determination was carried out with a Litesizer 500 Anton Paar equipment, with the following specifications: measuring range ≥1000 mV, mobility range 10^−11^–2 × 10^−7^ m^2^ V^−1^ s^−1^, 1000 scans, and at 25 °C.

##### SEM

SEM images were obtained on a JEOL JSM‐7800F Prime instrument at an accelerating voltage of 5 and 15 kV. All the samples were dried in air and sputtered with gold to 5 nm thickness using a sputter coater (Leica EM ACE 600).

##### Contact Angle Measurements

Contact angle measurements with Milli‐Q water were performed using a Theta Optical Tensiometer (Biolin Scientific). Each measurement was recorded over a duration of 80 s.

##### Tensile Testing

The mechanical tensile tests were performed using an Instron 5565 equipped with a 5 kN load cell and pneumatic clamps working at a pressure of 5 bars. The measurements were carried out using a gauge length of 15 mm and an extension rate of 9 mm min^−1^ (adopted from D3822 ASTM standard). The cotton threads samples after application of the consolidants were dried at room temperature for 24 h before tensile testing, each measurement was performed on seven samples.

##### Color Measurements

According to changes of the canvas before and after the dispersion treatment were evaluated using a portable spectrophotometer (Konica MinoltaCM‐700D) with a 3 mm diameter target area, analyzed through CM‐S100w (SpectraMagicNX) software according the CIELAB color space. Measurements were taken at five different areas of the sample placed on a black backing sheet.

## Conflict of Interest

The authors declare no conflict of interest.

## Supporting information

Supplementary Material

## Data Availability

The data that support the findings of this study are available from the corresponding author upon reasonable request.

## References

[1] G. Hedley , Stud. Conserv. 1988, 33, 133.

[2] N. Ryder , Conservator 1986, 10, 31.

[3] A. Fornari , M. Rossi , D. Rocco , L. Mattiello , Appl. Sci. 2022, 12, 12846.

[4] M. Oriola‐Folch , G. Campo‐Francés , A. Nualart‐Torroja , C. Ruiz‐Recasens , I. Bautista‐Morenilla , Herit. Sci. 2020, 8, 23.

[5] N. Böhme , M. Anders , T. Reichelt , K. Schuhmann , A. Bridarolli , A. Chevalier , Herit. Sci. 2020, 8, 1.

[6] O. Nechyporchuk , K. Kolman , A. Bridarolli , M. Odlyha , L. Bozec , M. Oriola , G. Campo‐Francés , M. Persson , K. Holmberg , R. Bordes , Carbohydr. Polym. 2018, 194, 161. 29801824 10.1016/j.carbpol.2018.04.020

[7] L. Spagnuolo , R. D’Orsi , A. Operamolla , ChemPlusChem 2022, 87, e202200204. 36000154 10.1002/cplu.202200204

[8] C. H. M. Camargos , G. Poggi , D. Chelazzi , P. Baglioni , C. A. Rezende , CS Appl. Nano Mater. 2022, 5, 13245.

[9] R. Giorgi , L. Dei , M. Ceccato , C. Schettino , P. Baglioni , Langmuir 2002, 18, 8198.

[10] R. Giorgi , C. Bozzi , L. Dei , C. Gabbiani , B. W. Ninham , P. Baglioni , Langmuir 2005, 21, 8495. 16114962 10.1021/la050564m

[11] G. Poggi , N. Toccafondi , L. N. Melita , J. C. Knowles , L. Bozec , R. Giorgi , P. Baglioni , Appl. Phys. A 2014, 114, 685.

[12] K. Kolman , O. Nechyporchuk , M. Persson , K. Holmberg , R. Bordes , Colloids Surf. A 2017, 532, 420.

[13] K. Kolman , O. Nechyporchuk , M. Persson , K. Holmberg , R. Bordes , ACS Appl. Nano Mater. 2018, 1, 2036.

[14] A. Bridarolli , M. Odlyha , O. Nechyporchuk , K. Holmberg , C. Ruiz‐Recasens , R. Bordes , L. Bozec , ACS Appl. Mater. Interfaces 2018, 10, 33652. 30149696 10.1021/acsami.8b10727

[15] T. Padfield , N. Padfield , D. S. H. Lee , A. Thøgersen , A. V. Nielsen , C. K. Andersen , M. Scharff , Herit. Sci. 2020, 8, 1.

[16] V. S. Saji , Colloids Surf. A 2020, 602, 125132.

[17] S. K. Fleetwood , S. Bell , R. Jetter , E. J. Foster , Soft Matter 2023, 19, 7020. 37676239 10.1039/d3sm00720k

[18] C. Li , Q. Liu , Z. Mei , J. Wang , J. Xu , D. Sun , J. Colloid Interface Sci. 2009, 336, 314. 19428022 10.1016/j.jcis.2009.03.080

[19] L. Lisuzzo , T. Hueckel , G. Cavallaro , S. Sacanna , G. Lazzara , ACS Appl. Mater. Interfaces 2020, 13, 1651. 33379868 10.1021/acsami.0c20443 PMC8021222

[20] A. Shirvani , S. A. H. Goli , J. Varshosaz , L. Salvia‐Trujillo , O. Martín‐Belloso , Food Bioproc. Tech 2023, 16, 1356. 10.1016/j.foodchem.2022.132934 35421652

[21] V. R. Gomes , O. J. Martinez‐Villabona , L. C. de Paiva , M. C. K. de Oliveira , L. R. Morantes , A. M. Percebom , Colloids Surf. A 2024, 688, 133619.

[22] L. Lisuzzo , G. Cavallaro , S. Milioto , G. Lazzara , Appl. Clay Sci. 2024, 247, 107217.

[23] S. Wang , K. Liu , X. Yao , L. Jiang , Chem. Rev. 2015, 115, 8230. 26244444 10.1021/cr400083y

[24] T. Darmanin , F. Guittard , Mater. Today 2015, 18, 273.

[25] T. Sheth , S. Seshadri , T. Prileszky , M. E. Helgeson , Nat. Rev. Mater. 2020, 5, 214.

[26] C. Solans , P. Izquierdo , J. Nolla , N. Azemar , M. J. Garcia‐Celma , Curr. Opin. Colloid Interface Sci. 2005, 10, 102.

[27] E. Nazarzadeh , T. Anthonypillai , S. Sajjadi , J. Colloid Interface Sci. 2013, 397, 154. 23452515 10.1016/j.jcis.2012.12.018

[28] M. Safaya , Y. C. Rotliwala , Mater. Today Proc. 2020, 27, 454.

[29] S. Saffarionpour , Food Eng. Rev. 2019, 11, 259.

[30] M. A. Vratsanos , W. Xue , N. D. Rosenmann , L. D. Zarzar , N. C. Gianneschi , ACS Cent. Sci. 2023, 9, 457. 36968532 10.1021/acscentsci.2c01194 PMC10037490

[31] N. L. Sitnikova , R. Sprik , G. Wegdam , E. Eiser , Langmuir 2005, 21, 7083. 16042427 10.1021/la046816l

[32] J. S. Komaiko , D. J. McClements , Compr. Rev. Food Sci. Food Saf. 2016, 15, 331. 33371595 10.1111/1541-4337.12189

[33] Z. S. Zeng , S. E. Taylor , Sep. Purif. Technol. 2020, 247, 116996.

[34] K. Thuresson , B. Lindman , J. Phys. Chem. B 1997, 101, 6460.

[35] B. Kronberg , K. Holmberg , B. Lindman , in Surface Chemistry Of Surfactants And Polymers, John Wiley & Sons, Hoboken 2014, pp. 278–282.

[36] W. Zhang , H. Xiao , L. Qian , Appl. Surf. Sci. 2014, 300, 80.

[37] P. G. Gan , S. T. Sam , M. F. Abdullah , M. F. Omar , J. Appl. Polym. Sci. 2020, 137, 48544.

[38] O. Nechyporchuk , K. Kolman , M. Oriola , M. Persson , K. Holmberg , R. Bordes , J. Cult. Herit. 2017, 28, 183.

[39] J. Wang , Y. Cao , G. Li , Exp. Fluids 2022, 63, 50.

