# Supplementary material for: Beeswax Nanoemulsion for Consolidation and Hydrophobization of Canvases
Source: Chempluschem. 2025 May 27;90(7):e202500058. doi: 10.1002/cplu.202500058 (PMC12261051; doi:10.1002/cplu.202500058)
Supplement: Supplementary file 1 — Supplementary Material [file CPLU-90-e202500058-s001.pdf]

Supporting Information

Beeswax Nanoemulsion for Consolidation and Hydrophobization of Canvases

Yiming Jia, [a] Krister Holmberg, [a] Romain Bordes*[a]

[a] Dr. Yiming Jia, Prof. Dr. Krister Holmberg, Prof. Dr. Romain Bordes
Department of Chemistry and Chemical Engineering, Applied Chemistry, Chalmers University of Technology, 412 96 Gothenburg, Sweden
E-mail: bordes@chalmers.se

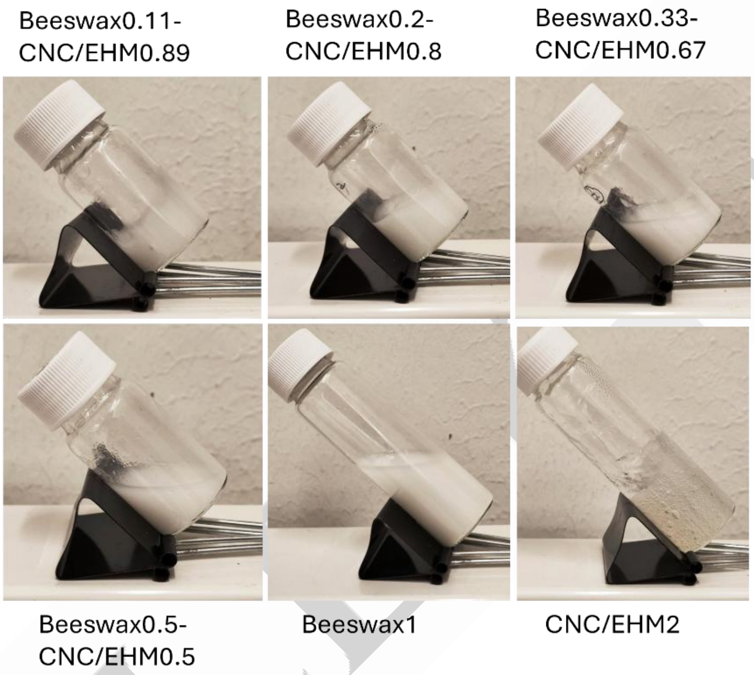

Figure S1. The photograph of the prepared formulations.

WILEY-VCH

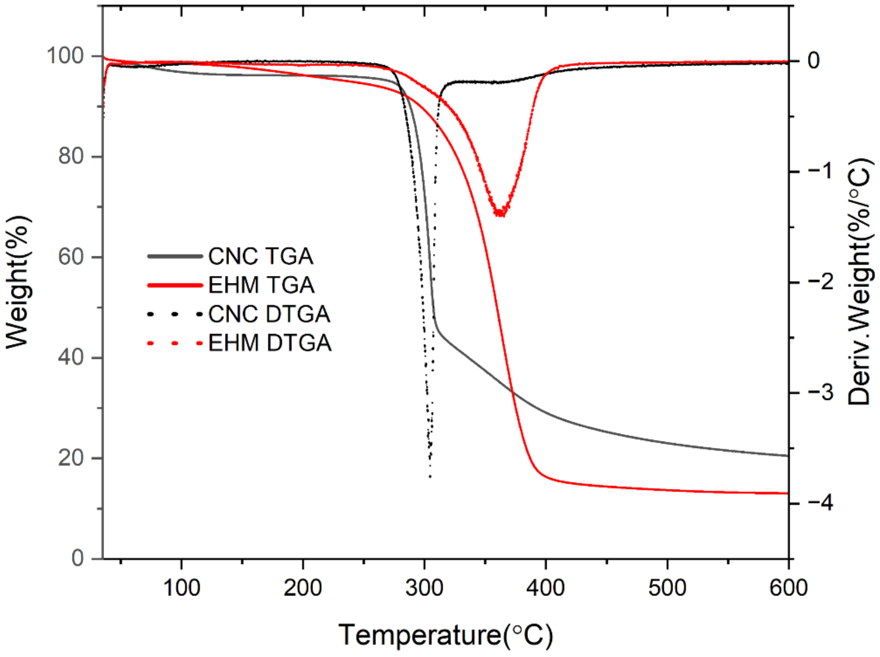

Figure S2. TGA (solid) and derivative thermogravimetry (dash) curves of CNC (black) and EHM (red).

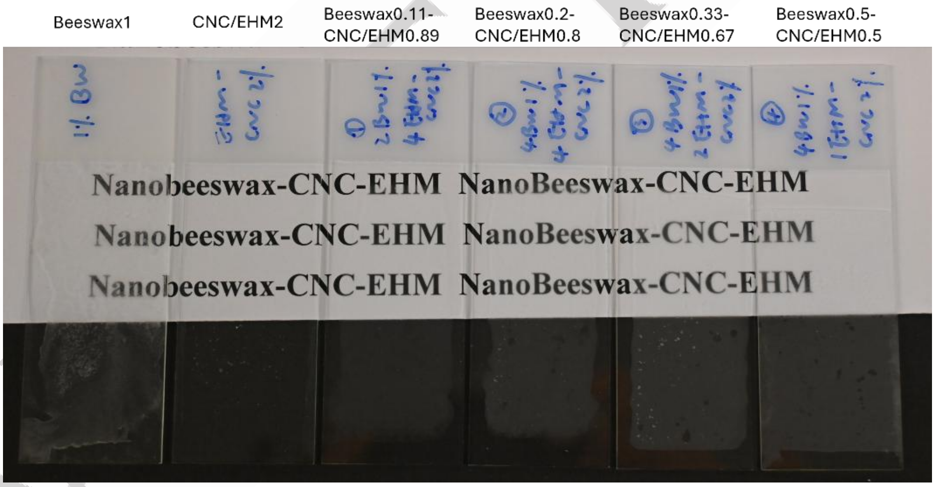

Figure S3. The photograph of the formulations after drying on glass slides.

Table S1. Nonlinear fitting of contact angle as a function of time for the formulations applied on degraded canvas.

Formulations	Fit of dynamic contact angle
CNC/EHM2	y = 123.069 -20.400 * exp (x * 0.217), R^2 = 0.9987
Beeswax0.11-CNC/EHM0.89	y = 107.896 -1.096 * exp (x * 0.224), R^2 = 0.9952
Beeswax0.2-CNC/EHM0.8	y = 112.920 -0.092 * exp (x * 0.185), R^2 = 0.9919
Beeswax1	y = 112.920 +24.518 * exp (-x * 0.055), R^2 = 0.9797
Beeswax0.33-CNC/EHM0.67	y = 125.068 +1.437 * exp (-x * 0.034), R^2 = 0.9710
Beeswax0.5-CNC/EHM0.5	y = 131.973 +0.626 * exp (-x * 0.056), R^2 = 0.8573

Table S2. The increased mass after treatment with the formulation on the degraded cotton square.

Formulation	Mass increase (wt%)
No-treatment	-
Beeswax1	0.17
CNC/EHM2	5.92
Beeswax0.11-CNC/EHM0.89	5.85
Beeswax0.2-CNC/EHM0.8	3.87
Beeswax0.33-CNC/EHM0.67	3.31
Beeswax0.5-CNC/EHM0.5	2.48

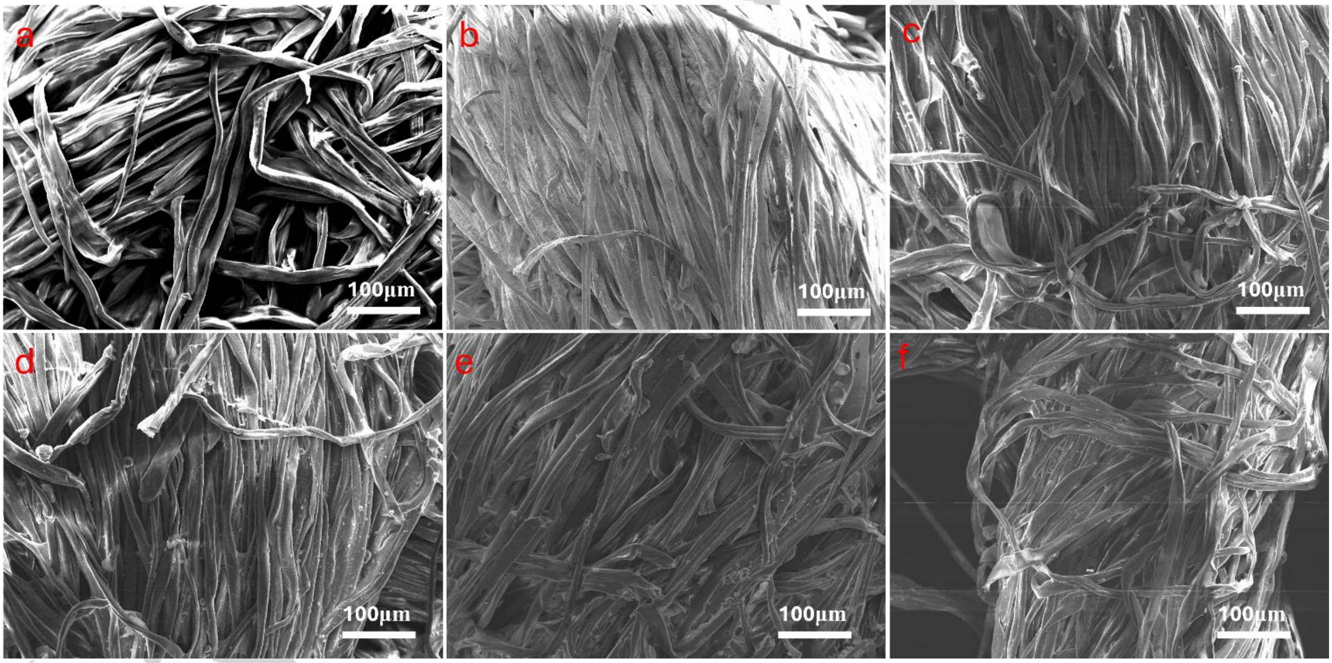

Figure S4. SEM image of degraded canvas after treatment with (a) CNC/EHM2, (b) beeswax1 (c) beeswax0.11-CNC/EHM0.89, (d) beeswax0.2-CNC/EHM0.8, (e) beeswax0.33-CNC/EHM0.67 and (f) beeswax0.5-CNC/EHM0.5.

WILEY-VCH
